# Psychometric properties of an Arabic translation of the long (12 items) and short (7 items) forms of the Violent Ideations Scale (VIS) in a non-clinical sample of adolescents

**DOI:** 10.1186/s12888-023-05465-6

**Published:** 2024-01-02

**Authors:** Feten Fekih-Romdhane, Diana Malaeb, Ecem Yakın, Fouad Sakr, Mariam Dabbous, Sami El Khatib, Sahar Obeid, Souheil Hallit

**Affiliations:** 1 The Tunisian Center of Early Intervention in Psychosis, Department of psychiatry “Ibn Omrane”, Razi Hospital, Manouba, 2010 Tunisia; 2 Faculty of Medicine of Tunis, Tunis El Manar University, Tunis, Tunisia; 3 College of Pharmacy, Gulf Medical University, Ajman, United Arab Emirates; 4 Centre d’Etudes Et de Recherches en Psychopathologie Et Psychologie de La Santé, Université de Toulouse-Jean Jaurès, UT2J, 5 Allées Antonio Machado, Toulouse, 31058 France; 5 School of Pharmacy, Lebanese International University, Beirut, Lebanon; 6 Department of Biomedical Sciences, School of Arts and Sciences, Lebanese International University, Bekaa, Lebanon; 7 Center for Applied Mathematics and Bioinformatics (CAMB), Gulf University for Science and Technology (GUST), Hawally, Kuwait; 8 Social and Education Sciences Department, School of Arts and Sciences, Lebanese American University, Jbeil, Lebanon; 9 School of Medicine and Medical Sciences, Holy Spirit University of Kaslik, P.O. Box 446, Jounieh, Lebanon; 10 Psychology Department, College of Humanities, Effat University, Jeddah, 21478 Saudi Arabia; 11 Applied Science Research Center, Applied Science Private University, Amman, Jordan

**Keywords:** Violent ideations, Violence, Violent ideations acale, Psychometric properties, Arabic

## Abstract

**Background:**

Accurately measuring violent ideations would be of particular high relevance in Arab countries, which are witnessing an unprecedented increase in violence rates among adolescents because of the widespread social, economic and political unrest in the region. Therefore, the present study aimed to test the psychometric properties of an Arabic translation of the 12-item and the 7-item forms of the Violent Ideations Scale (VIS and VIS-SF) in a sample on non-clinical Arabic-speaking adolescents.

**Methods:**

Five hundred seventy-seven community adolescents (mean age of 15.90 ± 1.73 years, 56.5% females) answered an anonymous online survey comprising an Arabic translation of the Violent Ideations Scale (VIS) and a measure of physical aggression.

**Results:**

Confirmatory factor analyses (CFA) offered support for the single-factor structure of the Arabic VIS and the VIS-SF. Both the long and short forms of the scale yielded excellent internal consistency, with McDonald’s ω coefficients of 0.96 and 0.94 and Cronbach’s α coefficients of 0.96 and 0.94, respectively. Multi-group CFA established measurement invariance across gender groups. Finally, results revealed significant and positive correlations between the two forms of the VIS and physical aggression scores, thus supporting concurrent validity.

**Conclusion:**

Both the VIS and VIS-SF have demonstrated good psychometric properties in their Arabic versions, and suitability for sound assessment of violent ideations. We therefore expect that these measures assist clinicians in risk assessment and management of violence, and help foster research in this area in Arab countries.

## Introduction

Violent ideations (VIs) refer to thoughts, fantasies or daydreams of inflicting physical or psychological harm on other people [1]. They may, but do not need to be, intrusive or ruminative in nature (e.g. [2]). VIs are distinct from aggressive delusions, threats or plans to commit an aggressive act, and do not involve ideations of self-directed and sexual violence (e.g. [1, 3]). A relatively limited amount of research has focused on VIs, especially on non-sexual violent fantasies and cognitions [4]. Available evidence suggests that mental images and thoughts with violent content are more frequent than commonly assumed, both in clinical [5] and non-clinical [6] populations. For instance, a study indicated that of 32.5% US young people at clinical high-risk for psychosis experienced VIs [5]. One of the first studies attempting to quantify VIs in the general public revealed that 68% of US undergraduate students acknowledged having had at least one homicidal ideation in their lifetime [6]. Another study found that 36% and 37% of youths in Switzerland reported having experienced at least once thoughts of taking violent revenge and humiliating someone they despised [1].

Recent years have seen a growing interest from scholars and clinicians in VIs as candidate indicators for future risk of violence and dangerousness, as core elements in the understanding of aggression and its prevention, as possible outcomes of mental health problems, and as potential treatment targets (e.g. [7–9]). Indeed, VIs have proven to be closely connected to a broad range of psychiatric conditions, including serious mental illnesses (i.e., schizophrenia, schizoaffective disorder, bipolar disorder, unipolar depression) [10], panic attacks [11], substance use [12], and suicidality [13]. Using factor analytic approaches, a study found that VIs clearly emerged as being correlated with, but distinct from, a range of mental health dimensions, supporting that they should be considered as a separate dimension including from aggression [9]. In addition, there is some limited evidence that VIs may be useful to predict potential risk for future interpersonal violent behaviors [1, 14, 15], especially in individuals with mental disorders [16]. As such, VIs were incorporated into some violence risk assessments [17], thus emphasizing their great usefulness from a clinical perspective. VIs have also been proposed as a target for intervention for violence reduction, with some promising preliminary evidence having been provided by a small pilot study among male violent offenders in England [7]. Furthermore, VIs are of potential relevance in research, as they play a determinant role in several psychological theories explaining violence, such as evolutionary theories of violence [18, 19], social cognitive theories of aggression [20], the general aggression model [2], theories focused on the role of self-control in aggression [21], and judgment and decision-making models [22]. Given the major theoretical and practical implications of VIs, validated measurement instruments are essential to violence theory development and assessment.

Prior research on VIs relied on single item measures (e.g., “I think about killing the people who have caused me problems”) [12, 23], adopted clinical interviews [24], or employed the Schedule of Imagined Violence [16]. However, all these methods may not achieve the potential advantages of reliability and cost-effectiveness of multi-item measures. As VIs are not necessarily associated with externalized behaviors, are of sensitive nature, and may be subject to social desirability bias [25], self-report measurement seems to be the most suitable, practical, and economical way of gathering information on those ideations or fantasies [26]. Few self-report measures currently exist that were designed specifically to assess VIs. The most frequently used measure is the Schedule of Imagined Violence (SIV, [16]), which consists of a set of eight structured questions, each examined separately. A first question (i.e. “Do you ever have daydreams or thoughts about physically hurting or injuring some other persons?”) is followed by seven other questions inquiring about further information on these VIs (e.g., recency, frequency, chronicity). Another measure is the Firestone Assessment of Violent Thoughts (FAVT, [27]), which assesses four types of angry affect and negative thoughts patterns (i.e., negative critical thoughts, thoughts of being disregarded, social mistrust, and thoughts of overt aggression). Nevertheless, the FAVT has unknown psychometric properties, and has mainly been used on small and predominantly male samples [28].

To bridge these gaps, Murray et al. [1] developed the Violent Ideations Scale (VIS) as a multi-item scale designed to ensure a brief and robust evaluation of VIs. An initial pool of 15 items was developed in the German language, to finally retain 12 items after removing three items on suicidal ideations and sexual VIs. All remaining items loaded into a single factor with small measurement differences between gender. The sensitivity demonstrated the power of the VIS to correctly classify over 70% of people who had previously engaged in criminal violence (i.e. robbery, extortion, assault, or carrying a weapon) using the 15.5 cut-off point obtained. Concurrent validity and content validity were attested through significant correlation with other violence-related structures in a sample of 1,276 community youth. The scale was then translated and validated from German to English by McKenzie et al. [29]. The English version showed adequate psychometric properties in a sample of 116 adults in terms of internal consistency, test–retest reliability, and concurrent validity. The VIS has then been validated in other countries and languages, including Spanish [30] and Chinese [31]. Later, a 7-item short form of the scale (VIS-SF) was developed among Chinese University Students, and was shown to be consistent with the full form in terms of factor structure, reliability and validity [31]. This short version carries the advantages of reducing administration time and burden, and decreasing research costs, while maintaining a good psychometric quality [31]. To date, there are no Arabic validated tools assessing VIs to the best of our knowledge. In addition, we could find no previous studies on VIs emerging from Arab countries.

### The present study

Items of the VIS are derived from Western culture, which is individual-centered [32], attaching great importance to individual freedom, needs, interests and rights. In contrast, Arab culture is group-oriented [33], which means that individual interests are rather based on collective interests thus favoring tolerance, compromise, avoiding inter-individual competition, promoting social values and interest of the group. In such cultural environments, individuals may be less prone to experience VIs, especially when their interests conflict with those of collectivity or bring no benefits for society (e.g., “beating up a stranger for no particular reason”) [31]. However, items that can be to some extent beneficial to the group (e.g., “killing someone who insulted my family or friends” or “using violence to get back at someone who harmed a person close to me”) are likely to be endorsed by Arab individuals. The concept of “blood revenge” rooted in the Arab culture [34] and reflected in the Islamic religion (“Al-Qisas” or retaliation) [35] is an example of how violence thoughts and behaviors can be driven by interests of the group. As broad cultural differences may impact the applicability and effectiveness of the VIS, psychometric studies are necessary to consolidate its validation and confirm its suitability for use in a given context. Furthermore, accurately measuring VIs would be of particular high relevance in Arab countries, which are witnessing an unprecedented increase in violence rates among adolescents because of the widespread social, economic and political unrest in the region [36]. The present study aimed to test the psychometric properties of an Arabic translation of the 12-item and the 7-item forms of the VIS in a sample on non-clinical Arabic-speaking adolescents. The following hypotheses were formulated: (a) the Arabic VIS and VIS-SF have a one-factor structure, consistently with previous versions; (b) Both versions of the scale show adequate internal consistency (alpha and omega values exceeding 0.7 [37]); (c) A good concurrent validity can be demonstrated against a measure of physical aggression. In particular, we expect to find significant and positive correlations between the two forms of the VIS and physical aggression scores.

## Methods

### Procedures

All data was collected via a Google Form link in July 2023. The research team approached people and asked them to fill the survey; those who accepted were asked to forward the link to other people they might know, explaining the snowball sampling technique followed. Inclusion criteria for participation included being of a resident and citizen of Lebanon and aged between 13 and 18 years. The “remove duplicates” option in excel ensured that no participant submitted the same responses twice; none was removed after this process. After providing digital informed consent, participants were asked to complete the anonymous survey. Participants completed the survey voluntarily and without remuneration [38].

### Participants

Five hundred seventy-seven participants filled the survey, with a mean age of 15.90 ± 1.73 years (age range 13–18 years) and 56.5% females.

### Measures

#### Demographics

Participants were asked to provide their demographic details consisting of age and gender.

#### The violent ideation scale long (SIV) and short (SIV-SF) forms

The forward and backward translation method was applied to the scale following international guidelines [39]. The English version was translated to Arabic by a Lebanese translator who was completely unrelated to the study. The Arabic translated version was proofread and edited by two research team members, who are native Arabic speakers from two Arab countries and regions (North African Tunisia and Middle Eastern Lebanon), to ensure that Arabic words have the same meaning across countries and that no outdated terms were used. Afterwards, a Lebanese psychologist with a full working proficiency in English, translated the Arabic version back to English. The initial and translated English versions were compared to detect and later eliminate any inconsistencies by a committee composed of the research team and the two translators [40, 41]. A pilot study was conducted on 30 adolescents before the start of the official data collection to make sure all questions are well understood; no changes were done consequently.

#### Physical aggression

The physical aggression subscale of the Buss–Perry Aggression Questionnaire-Short Form (BPAQ-SF) [42], which is composed of three items (e.g., “I have threatened people I know”) was used in the present study. Each item is rated on a 5-point Likert scale. Higher scores indicate higher levels of physical aggression (ω = 0.83 / α = 0.82).The Arabic validated version yielded good psychometric properties [43], the BPAQ-SF is a short version of the BPAQ, and it contains 12 items.

### Analytic Strategy

#### Confirmatory factor analysis

There were no missing responses in the dataset. Confirmatory factor analysis (CFA) was conducted on the whole sample (N = 577) to test the original one-factor structure of the VIS [1], and if divergent apply the exploratory-to-confirmatory factor analysis strategy. The CFA was performed using RStudio (Version 1.4.1103 for Macintosh) [44] and the Lavaan [45] and semTools [46] packages. As this scale uses a 4-point Likert scale, following the original validation study [1], we used Weighted Least Squares with Mean and Variance (WLSMV) estimation method, which is known to be more appropriate for ordinal data [47]. Also, following the methodology applied in the original validation study [1], we reported and considered the CFA model to fit well if the Tucker–Lewis index (TLI) and the Comparative Fit Index (CFI) were > 0.90, the Root Mean Square Error of Approximation (RMSEA) was < 0.08, and the Standardized Root Mean Square Residual (SRMR) was < 0.08 [48]. The average variance extracted (AVE) was used as evidence of convergent validity, with values of ≥ 0.50 considered adequate [49].

#### Gender invariance

To examine gender invariance of VIS scores, we conducted multi-group CFA [50] using the total sample. Measurement invariance was assessed at the configural, metric, and scalar levels [51]. We accepted ΔCFI ≤ 0.010 and ΔRMSEA ≤ 0.015 or ΔSRMR ≤ 0.010 as evidence of invariance [50].

#### Reliability analysis and validity

Composite reliability was assessed using McDonald’s ω and Cronbach’s α [52], with values greater than 0.70 reflecting adequate composite reliability. The VIS scores were considered normally distributed according to their skewness (= 1.202) and kurtosis (= 0.475) values varying between ± 1.96. Consequently, the Pearson test was used to correlate those scores with physical aggression, self-esteem and wellbeing. The Student *t* test was used to compare VIS scores between genders.

## Results

A one-factor confirmatory analysis was conducted to test the factor structure of the VIS-12 found in the original validation study [1]. This model showed a satisfactory fit with a high CFI of 0.957, a high TLI of 0.947, a SRMR of 0.034, SBχ²/df = 133.62/66 = 2.02 and good RMSEA of 0.051 90% CI [0.040, 0.061]. The AVE value was adequate (= 0.56). The McDonald’s ω and Cronbach’s α values were excellent as well (0.96 and 0.96 respectively).

Similar results were found for the VIS-SF-7 model as follows: This model showed a satisfactory fit with high CFI of 0.973, a high TLI of 0.965, a SRMR of 0.030, SBχ²/df = 72.39/45 = 1.61 and good RMSEA of 0.043 90% [CI 0.029, 0.057]. The AVE value was adequate (= 0.62). The McDonald’s ω and Cronbach’s α values were excellent as well (0.94 and 0.94 respectively). Factor loadings are can be found in Table 1.

**Table 1:** Descriptive statistics, category response distributions and factor loadings derived from the confirmatory factor analysis of the one-factor model of the VIS and VIS-SF in the total sample

Items	Never	Rarely	Sometimes	Often	Very often	Mean (SD)	Factor loading (VIS, N=…)	Factor loading (VIS-SF, N=…)
1. … killing someone I know	411 (71.2%)	71 (12.3%)	76 (13.2%)	17 (2.9%)	2 (0.3%)	1.49 (0.86)	0.71	0.70
2. … using violence to get back at someone who harmed me	321 (55.6%)	104 (18.0%)	111 (19.2%)	31 (5.4%)	10 (1.7%)	1.80 (1.04)	0.78	0.80
3. … severely injuring someone I dislike	354 (61.4%)	97 (16.8%)	91 (15.8%)	28 (4.9%)	7 (1.2%)	1.68 (0.99)	0.82	0.82
4. … beating up a stranger for no particular reason	407 (70.5%)	71 (12.3%)	73 (12.7%)	19 (3.3%)	7 (1.2%)	1.52 (0.92)	0.73	
5. … killing someone who insulted my family or friends	374 (64.8%)	82 (14.2%)	83 (14.4%)	30 (5.2%)	8 (1.4%)	1.64 (1.00)	0.78	0.80
6. … humiliating someone I despise	339 (58.8%)	105 (18.2%)	99 (17.2%)	26 (4.5%)	8 (1.4%)	1.72 (0.99)	0.80	
7. … killing a person close to me who humiliated or offended me	405 (70.2%)	63 (10.9%)	83 (14.4%)	20 (3.5%)	6 (1.0%)	1.54 (0.93)	0.78	0.77
8. … humiliating someone weaker than me	405 (70.2%)	74 (12.8%)	75 (13.0%)	20 (3.5%)	3 (0.5%)	1.51 (0.88)	0.71	
9. … using violence to get back at someone who harmed a person close to me	356 (61.7%)	84 (14.6%)	99 (17.2%)	31 (5.4%)	7 (1.2%)	1.70 (1.01)	0.87	0.87
10. … beating up someone I find totally repulsive	376 (65.2%)	101 (17.5%)	79 (13.7%)	17 (2.9%)	4 (0.7%)	1.57 (0.88)	0.75	0.76
11. … causing someone intense pain	375 (65.0%)	83 (14.4%)	85 (14.7%)	29 (5.0%)	5 (0.9%)	1.62 (0.97)	0.83	
12. … beating someone to a pulp because they made me truly angry	397 (68.8%)	78 (13.5%)	69 (12.0%)	27 (4.7%)	6 (1.0%)	1.56 (0.95)	0.81	

### Measurement invariance across gender

Similar to the original validation study, this one-factor model was further used as the basis for assessing gender invariance for the scale. Model fit for configural, metric and scalar invariance is provided in Table 2. No significant difference was found between males and females in terms of VIS scores (19.98 ± 9.75 vs. 18.85 ± 9.41, *t*(575) = 1.408, *p* = .160) and VIS-SF scores (11.83 ± 5.79 vs. 11.09 ± 5.62, *t*(575) = 1.552, *p* = .121).

**Table 2:** Fit indices of the original and measurement invariance across gender models in the total sample for the VIS and VIS-SF scales

MODEL (12-İTEM)	Robust TLI	Robust CFI	Robust RMSEA (low-UP)	SRMR	SBχ²	df	Model Comparison	ΔRobust CFI	ΔRobust TLI	ΔSBχ²	Δ*df*	Pr(> Chisq)
Whole sample (N = 577)	0.947	0.957	0.051 (0.040–0.061)	0.034	133.623	66						
Config-model	0.973	0.974	0.036 (0.022–0.049)	0.048	71.241	130	Configural vs. metric	0.000	0.002	13.905	11	0.238
Metric-model	0.971	0.974	0.038 (0.023–0.050)	0.047	66.560	119	Metric vs. scalar	0.000	0.002	13.905	11	0.238
Scalar-MODEL	0.973	0.974	0.036 (0.022- 0. 049	0.048	71.241	130						
**MODEL** **(7-İTEM)**	**Robust TLI**	**Robust CFI**	**Robust RMSEA (low-UP)**	**SRMR**	**SBχ²**	**df**	**Model Comparison**	**ΔRobust CFI**	**ΔRobust TLI**	**ΔSBχ²**	**Δ** ***df***	**Pr(> Chisq)**
Whole sample (N = 577)	0.957	0.972	0.061 (0.041–0.082)	0.030	72.391	40						
Config-model	0. 962	0.964	0.058 (0. 039 − 0. 077)	0.049	30.245	40	Configural vs. metric	0.003	0.003	10.982	6	0.08895 (Not-sig)
Metric-model	0. 0.959	0. 967	0.060 (00.40-0.081)	0.047	26.481	34	Metric vs. scalar	0.002	0.003	10.982	6	0.08895 (Not-sig)
Scalar-MODEL	0.962	0.964	0.058 (0.039–0.077)	0.049	30.245	40						

*Note*: CFI = Comparative Fit Index; RMSEA = Steiger-Lind Root Mean Square Error of Approximation; SRMR = Standardised Root Mean Square Residual; SBχ² = Satorra-Bentler Chi-square

### Concurrent validity

Higher VIS (*r* = .59; *p* < .001) and VIS-SF (*r* = .59; *p* < .001) scores were significantly associated with more physical aggression.

## Discussion

This study aimed to provide, for the first time, a robust measure of VIs for use among Arabic-speaking individuals. Findings provided support for the factorial validity, appropriate reliability coefficients, and good composite reliability of the Arabic VIS, both in its long and short forms. This suggests that the two versions of the VIS are suitable for use in Arab youth. As the VIS-SF showed adequate psychometric qualities while requiring fewer items, it may be a desirable alternative for large-scale and multiple-point future studies. However, clinicians and researchers should bear in mind two important points regarding the applicability of these scales. First, the 12-item VIS does not contain items on self-violence (i.e., thoughts of self-injury and suicide) and sexual violence. It is, therefore, not appropriate for assessing these aspects. Second, all 7 items of the VIS-SF are exclusively about physical interpersonal VIs. Therefore, aside from the length of the scale, the choice of one or the other version of the VIS should depend on the actual need and purpose.

Prior psychometric findings on the VIS that mainly emerged from Western countries may be non-generalizable to all human population [53]. Psychometric studies are therefore warranted to test whether the structures of the Arabic versions of the VIS/VIS-SF are consistent with those found in Western and Chinese research, and whether the scales apply to people who grew up in an Arab cultural background. In the present study and sample, CFA offered support for the single-factor structure of the VIS and the VIS-SF, further suggesting that VIs is likely a unidimensional construct. Previous examinations of the loadings of the one-factor solution of both long and short versions revealed acceptable magnitudes and significant loadings on the single factor for all items in different samples and languages (i.e., original scale German [1], Spanish [30], English [29], Chinese [31]). The unidimensional structure seems to hold up across cultures. It enables an easy calculation of a global score to derive a single assessment reflecting the continuum of VIs levels, thus fostering its application in screening studies.

The Arabic VIS and VIS-SF yielded excellent internal consistency, with McDonald’s ω coefficients of 0.96 and 0.94, and Cronbach’s α coefficients of 0.96 and 0.94, respectively. It is of note that internal consistency was not calculated in the original validation study [1]. Later validations were able to demonstrate good reliability of the Spanish (α value of 0.94 and ω value of 0.92; [30]), English (α value of 0.94; [29]), Chinese long (α value of 0.91 and ω value of 0.92; [31]) and short (α and ω values of 0.90; [31]) forms of the scale. This adds to the evidence of reliability of the VIS and VIS-SF. More psychometric studies in other cultural contexts are required to confirm the factorial validity of the originally proposed unidimensional model of the VIS.

Furthermore, multi-group analyses established measurement invariance across gender, which is inconsistent with Murray et al.’s findings that item functioning on the VIS differed slightly between German-speaking male and female participants [1]. In particular, minor measurement differences were found between males and females in Item 3 referring to violent revenge, with males having a higher intercept than females. The primary distinction between the model in both gender groups was that for a female and male of the same latent trait grade, a female would be expected to score lower on Item 3 and be less willing to experience violent retaliation-related VIs than females [1]. In Arab culture, however, violent retaliation tends to be shared by the social group as a whole, and may be not necessarily linked to the norm of masculinity. Similar to our findings, the Chinese [31] and the Spanish [30] studies evidenced cross-gender invariance. However, in the Spanish study, authors have been led to collapse categories to perform a gender invariance analysis due to the small number of female participants reporting certain VIs [30], which can limit conclusions regarding this psychometric property. Meeting measurement invariance across gender groups has important implications for the researchers for interpretations of the VIS scores, as it means that the VIS and VIS-SF reflect the same construct which is measured in the same way across males and females. This means that differences in latent scores are attributable to real differences in the VIs levels, and not to variations in understanding or responding to items between male and female respondents [54].

The original validation of the VIS found that VIs scores significantly and positively correlated with self-control and aggressive behavior [1]. Likewise, good concurrent validity of the VIS was demonstrated in the English-speaking [29], and the Chinese-speaking [31] samples by using the BPAQ-SF. In the Spanish-speaking sample, authors considered aggressive behaviors committed in the past month (assessed by six items of the EBIP-Q—European Bullying Intervention Project Questionnaire [55]) as indicators of concurrent validity of the VIS. In the present study, self-reported physical aggression assessed by the AQ was also used as a measure to test criterion validity. Results revealed significant and positive correlations between the two forms of the VIS and physical aggression scores. This broadly corroborates previous findings and assumptions that the VIS allows for effectively distinguishing individuals with more VIs, who also can be at increased risk of future violence. Based on the present findings and previous literature, we cautiously suggest that the VIS could assist clinicians in identifying at-risk groups and intervene with them to reduce the likelihood of committing violent acts [31].

### Study limitations and future research perspectives

This study is limited by the cross-sectional design. Future longitudinal experimental research is needed to further explore the predictive validity of the SIV and the SIV-SF on aggressive and violent behaviors. The reliance on self-report measures represents another limitation, as it may lead to response, recall and social desirability biases. The representativeness of our sample to the wider adolescent population may be limited by the online recruitment method. The sample consisted of non-clinical adolescents, which may limit the generalizability to clinical samples. The examination of the Arabic VIS psychometric properties was performed based on a sample from a single country and culture. Future validation studies may extend the sample to include participants from Arab countries and cultural backgrounds other than Lebanon. In addition, data on participants’ personal psychiatric history was not collected, and participants were not investigated using clinical interviews. Limited by the length of our survey, we only used the physical aggression subscale of the BPAQ-SF to examine concurrent validity of the VIS. Other important psychometric characteristics were not explored in the context of the present study, such as test-retest reliability. Finally, researchers still need to examine measurement invariance for language (Arabic versus original German or English versions of the VIS), to allow meaningful comparisons of findings from investigations in Arabic-speaking and other linguistic-speaking groups.

## Conclusion

Because of the substantial relevance of VIs as a core feature of the emotional and neurocognitive processes linked to violence, and since Arab youth are experiencing high rates of violence over the last years, there is a need to provide valid and reliable measures of VIs for use in Arab cultural contexts. In an effort to address this yet unmet need, the current study sought to test the psychometric characteristics of the 12-item and the 7-item forms of the VIS among Arabic-speaking youth. Both forms have demonstrated good psychometric properties, and suitability for sound assessment of VIs. We therefore expect that these measures assist clinicians in risk assessment and management of violence, and help spark future research in this area in Arab countries.

## Data Availability

The datasets generated and/or analysed during the current study are not publicly available due to restrictions from the ethics committee but are available from the corresponding author on reasonable request.

## References

[1] Murray AL, Eisner M, Ribeaud D (2018). Development and validation of a brief measure of violent thoughts: the violent Ideations Scale (VIS). Assessment.

[2] DeWall CN, Finkel EJ, Denson TF (2011). Self-control inhibits aggression. Soc Pers Psychol Compass.

[3] Gellerman DM, Suddath R (2005). Violent fantasy, dangerousness, and the duty to warn and protect. J Am Acad Psychiatry Law Online.

[4] Gilbert F, Daffern M (2017). Aggressive scripts, violent fantasy and violent behavior: a conceptual clarification and review. Aggress Violent Beh.

[5] Brucato G (2019). Prevalence and phenomenology of violent ideation and behavior among 200 young people at clinical high-risk for psychosis: an emerging model of Violence and psychotic Illness. Neuropsychopharmacology.

[6] Kenrick DT, Sheets V (1993). Homicidal fantasies. Ethol Sociobiol.

[7] Akerman G (2008). The development of a fantasy modification programme for a prison-based therapeutic community. Int J Therapeutic Communities.

[8] Monahan J (2000). Developing a clinically useful actuarial tool for assessing Violence risk. Br J Psychiatry.

[9] Murray AL (2017). Situating violent ideations within the landscape of mental health: associations between violent ideations and dimensions of mental health. Psychiatry Res.

[10] Roché MW (2018). Prevalence and risk of violent ideation and behavior in Serious Mental illnesses: an analysis of 63,572 patient records. J Interpers Violence.

[11] Korn ML, Plutchik R, Van Praag HM (1997). Panic-associated suicidal and aggressive ideation and behavior. J Psychiatr Res.

[12] Bruns D, Disorbio JM, Hanks R (2007). Chronic pain and violent ideation: testing a model of patient Violence. Pain Med.

[13] Brent DA (1993). Psychiatric risk factors for adolescent Suicide: a case-control study. J Am Acad Child Adolesc Psychiatry.

[14] Anderson CA, Bushman BJ (2002). Human aggression. Ann Rev Psychol.

[15] Sturup J, Monahan J, Kristiansson M (2013). Violent behavior and gender of Swedish psychiatric patients: a prospective clinical study. Psychiatric Serv.

[16] Grisso T (2000). Violent thoughts and violent behavior following hospitalization for mental disorder. J Consult Clin Psychol.

[17] Monahan J (2005). An actuarial model of Violence risk assessment for persons with mental disorders. Psychiatric Serv.

[18] Duntley JD, Buss DM (2011). Homicide adaptations. Aggress Violent Beh.

[19] Eisner M (2009). The uses of Violence: an examination of some cross-cutting issues. Int J Confl Violence (IJCV).

[20] Anderson CA, Huesmann LR. *Human aggression: A social-cognitive view* The Sage handbook of social psychology, 2007: p. 259–287.

[21] Denson TF (2011). Understanding impulsive aggression: angry rumination and reduced self-control capacity are mechanisms underlying the provocation-aggression relationship. Pers Soc Psychol Bull.

[22] Van Gelder J-L (2013). Beyond rational choice: the hot/cool perspective of criminal decision making. Psychol Crime Law.

[23] Bruns D, Disorbio JM (2000). Hostility and violent ideation: physical rehabilitation patient and community samples. Pain Med.

[24] Feng X (2019). Amygdalar volume and violent ideation in a sample at clinical high-risk for psychosis. Psychiatry Research: Neuroimaging.

[25] Piedmont R. *Social desirability bias* Encyclopedia of quality of life and well-being research, 2014: p. 6036–6037.

[26] Demetriou C, Ozer B, Essau C. *Self-Report Questionnaires. The Encyclopedia of Clinical Psychology, November 2017, 1–6*. 2015.

[27] Doucette-Gates A, Firestone RW, Firestone LA. Assessing violent thoughts: the relationship between thought processes and violent behavior. Psychologica belgica; 1999.

[28] Howden S, Midgley J, Hargate R (2018). Violent offender treatment in a medium secure unit. J Forensic Pract.

[29] McKenzie K (2021). Validation of the English language version of the violent Ideations Scale. J Interpers Violence.

[30] Urruela C et al. *Validation of the violent Ideations Scale (VIS) in Spain*. Int J Offender Ther Comp Criminol, 2023: p. 306624x221148126. 10.1177/0306624X221148126 36644834

[31] Xie M, Dai B. *Evaluation of the psychometric properties of the violent ideations scale and construction of a short form among Chinese University Students* Current Psychology, 2022.

[32] Hofstede G, Hofstede GJ, Minkov M. *Cultures and Organizations: Software of the Mind McGraw-Hill Education* Interpretation Handbook and Standard. Distilling the Essence (2005).(ed. by Fiona Colquhoun), Department of Conservation, Wellington, 2010.

[33] !!!. INVALID CITATION !!! [59, 60].

[34] Aliyev H, Souleimanov EA. Fighting against Jihad? Blood revenge and anti-insurgent mobilization in Jihadist Civil Wars. Studies in Conflict & Terrorism; 2022. pp. 1–21.

[35] El-Awa MAS. The theory of punishment in Islamic law: a comparative study. SOAS University of London; 1972.

[36] Makhlouf Obermeyer C. *Adolescents in Arab countries: health statistics and social context* DIFI Family Research and Proceedings, 2015. 2015(1): p. 1.

[37] Viladrich C, Angulo-Brunet A, Doval E (2017). A journey around alpha and omega to estimate internal consistency reliability. Anales De psicología.

[38] Swami V (2022). Psychometric properties of an arabic translation of the functionality appreciation scale (FAS) in Lebanese adults. Body Image.

[39] Beaton DE (2000). Guidelines for the process of cross-cultural adaptation of self-report measures. Spine (Phila Pa 1976).

[40] Fekih-Romdhane F et al. *Psychometric Properties of an Arabic Translation of the Multidimensional Social Support Scale (MSPSS) in a community sample of Lebanese Adults* 2022. 10.1186/s12888-023-04937-z PMC10265861 37316897

[41] Hallit S et al. *Validation of the Arabic Version of the Freiburg Mindfulness Inventory (FMI-Ar) Among a Sample of Lebanese University Students* 2022.

[42] Bryant FB, Smith BD (2001). Refining the architecture of aggression: a measurement model for the Buss–Perry Aggression Questionnaire. J Res Pers.

[43] Fekih-Romdhane F (2023). Association between bullying victimization and aggression in Lebanese adolescents: the Indirect Effect of repetitive negative Thinking—A path analysis Approach and scales Validation. Children.

[44] *R Core Team (2020). R: A language and environment for statistical computing. R Foundation for Statistical Computing, Vienna, Austria. URL *https://www.R-project.org/

[45] Rosseel Y. *lavaan: An R Package for Structural Equation Modeling. Journal of Statistical Software, 48(2), 1–36. URL *i>http://www.jstatsoft.org/v48/i02/ 2012.

[46] Jorgensen TD et al. *semTools: Useful tools for structural equation modeling. R package version 0.5-4. Retrieved from*: https://CRAN.R-project.org/package=semTools 2021.

[47] Li CH (2016). Confirmatory factor analysis with ordinal data: comparing robust maximum likelihood and diagonally weighted least squares. Behav Res Methods.

[48] Hu Lt, Bentler PM (1999). Cutoff criteria for fit indexes in covariance structure analysis: conventional criteria versus new alternatives. Struct Equation Modeling: Multidisciplinary J.

[49] Malhotra N, et al. Marketing research: an applied orientation. Deakin University; 2006.

[50] Chen FF (2007). Sensitivity of goodness of fit indexes to lack of measurement invariance. Struct Equation Modeling: Multidisciplinary J.

[51] Vadenberg R, Lance C (2000). A review and synthesis of the measurement in variance literature: suggestions, practices, and recommendations for organizational research. Organ Res Methods.

[52] Malkewitz CP (2023). Estimating reliability: a comparison of Cronbach’s α, McDonald’s ωt and the greatest lower bound. Social Sci Humanit Open.

[53] Henrich J, Heine SJ, Norenzayan A (2010). The weirdest people in the world?. Behav Brain Sci.

[54] Putnick DL, Bornstein MH (2016). Measurement invariance conventions and reporting: the state of the art and future directions for psychological research. Dev Rev.

[55] Ortega-Ruiz R, Rey RD, Casas JA (2016). Evaluar El bullying Y El cyberbullying validación española del EBIP-Q y Del ECIP-Q. Psicología Educativa.

